# Supporting comprehension: The advantages of multiple–choice over true—false practice tests

**DOI:** 10.3758/s13421-025-01726-9

**Published:** 2025-06-09

**Authors:** Lena Hildenbrand, Jennifer Wiley

**Affiliations:** Department of Psychology, University of Illinois at Chicago, 1007 W Harrison St MC 285, Chicago, IL 60607 USA

**Keywords:** Comprehension, Practice testing, Test format effects

## Abstract

While work on improving comprehension has primarily focused on open-ended generative activities, closed-ended practice tests using inference-type questions may also benefit understanding from text. Four experiments were designed to investigate how practice tests, specifically in multiple-choice and true—false formats, may support comprehension. Experiments 1 and 2 compared the two practice test formats to rereading. Both formats improved performance on a final essay test in Experiment 1, but in Experiment 2, only multiple-choice practice enhanced performance on a short-answer (SA) test. Experiment 3 introduced feedback on practice tests, but found no added benefit on the final SA test, which remained consistently better for those who completed the multiple-choice as compared with the true—false version of the practice test. Finally, manipulating text availability during practice tests in Experiment 4 improved performance on the final SA test. However, multiple-choice practice consistently led to better SA performance than true—false, regardless of text availability. The present work illustrates that the benefits from a closed-ended practice test with multiple-choice questions can persist over a delay and transfer to a set of new comprehension questions. At the same time, the results also highlight important constraints in that subtle nuances in question design can impact the observed benefits of practice testing on learning outcomes.

Decades of research on the *testing effect* have demonstrated that taking a practice test on to-be-learned materials can result in better learning than other nontesting activities such as simple restudy (Adesope et al., 2017; Dunlosky et al., 2013; Roediger & Karpicke, 2006; Rowland, 2014). While testing effects have been replicated many times and across a variety of different learning materials (Carpenter, 2009; Carpenter & DeLosh, 2006; Carpenter et al., 2006; Carrier & Pashler, 1992; Pan & Rickard, 2017; Pyc & Rawson, 2010; Rawson & Dunlosky, 2011; Roediger & Karpicke, 2006; Rowland, 2014; Zaromb & Roediger, 2010), some have argued the benefits of testing may be more limited as the complexity of the learning task increases (van Gog & Sweller, 2015). Alternatively, others suggest that the efficacy of testing for learning outcomes is not limited by the demands of the learning task but rather depends on how the testing experience engages the learner during practice (Karpicke & Aue, 2015). For simple tasks such as learning paired associates or wordlists, elaboration and retrieval effort/difficulty have been found to be common predictors for the degree to which testing activities might benefit memory (Carpenter, 2009; Carpenter & DeLosh, 2006; Carrier & Pashler, 1992; Kang et al., 2007; Pyc & Rawson, 2010).

For more complex tasks such as learning from lectures, videos, or assigned readings, learning outcomes may be measured in a variety of ways, including memory for the materials or by assessing comprehension or understanding of the lessons. Here, an important determinant for whether practice testing benefits learning might be the match between the processing demands at practice and the level at which actual learning will be assessed later (Hinze et al., 2013).

Learning from text is more complex than learning word lists in that learners must process text at multiple levels of representation to achieve comprehension (Kintsch, 1988). These levels range from shallow, more surface-level processing to deeper, more constructive processing in developing an understanding of the situation being described by the text. In the literature on text comprehension distinctions are made between three levels of processing. At the surface level the text is represented in the exact words as they appear. The text-base level then represents the propositional contents of the text. Finally, the situation-model level captures the implicit relations between ideas within a text as well as their connections with prior knowledge. Thus, to have really learned from a text is not limited to having memory of the information as it was presented but requires the learner to construct a coherent situation model or mental model of what the text is about (Kintsch, 1994). Surface-level processing is typically measured using memory-based questions that require the learner to reproduce information from the text, while understanding or comprehension is measured using inference questions that prompt learners to verify implicit relations between ideas within a text or apply the information from the text to novel situations (Wiley et al., 2005).

## Practice testing and text-based learning

The existing literature supports the idea that practice testing enhances text-based learning (Brabec et al., 2021; Butler, 2010; Butler & Roediger, 2008; Hinze et al., 2013; Kang et al., 2007; Karpicke, & Roediger, 2007; Little & Bjork, 2015; Little et al., 2012; Smith & Karpicke, 2014). However, this prior work on learning from text has varied along several dimensions. Much of the prior research on retrieval practice has focused on memory for text such as recall of facts or definitions explicitly stated in the text. The most robust testing effects are found when the same or similar items are used on the practice test and final test, and when feedback is included as part of the practice test activity (Butler & Roediger, 2008; Roediger & Karpicke, 2006; Smith & Karpicke, 2014). There are also some studies that have found benefits on new or related questions that were not previously tested. However, this body of research primarily addresses improvements in memory retention rather than comprehension (Brabec et al., 2021; Little & Bjork, 2015; Little et al., 2012).

For improving higher-level comprehension, evidence suggests that only practice test questions that prompt constructive processing enhance learning outcomes (Hinze et al., 2013). To foster constructive processing, studies on comprehension have prioritized open-ended, generative tasks, such as summarization or self-explanation (Chi et al., 1994; Davey & McBride, 1986; McNamara, 2004). When used for practice tests, these tasks require students to actively reconstruct knowledge, integrate relevant concepts, and articulate understanding in a meaningful way. In contrast, there is a concern that closed-ended testing formats—including multiple-choice and true—false questions—may only provide limited opportunities for this constructive processing as answer options are already provided rather than generated or “constructed” by the learner.

Nevertheless, closed-ended practice tests have been included as part of classroom learning activities where they have been shown to facilitate learning. However, in applied settings, these activities are often confounded with other instructional interventions, such as providing feedback (Pyburn et al., 2014) or having students discuss questions with each other (Smith et al., 2009). They often test for memory for information from the learning materials and may also fail to include a true no-testing control group (Bjork et al., 2014). In these studies, it is unclear how much of the benefit for learning is the direct result from the practice test alone. It remains uncertain whether benefits from closed-ended practice tests would extend to improving comprehension from self-guided reading-based tasks, which may engage different cognitive processes than improving memory for information. Further, no studies to date have directly compared different closed-ended question formats, such as multiple-choice versus true—false, in terms of their impact on text-based comprehension. Thus, research is needed to explore how practice testing with closed-ended questions affects *comprehension from text* and whether variations in question format influence these outcomes.

## Multiple-choice versus true—false practice tests

Given the lack of empirical evidence, a thought experiment reflecting on differences between the multiple-choice and true—false response formats suggests several distinctions that could influence their efficacy as tools for learning when included as part of practice tests. Probably the most obvious difference between them lies in how information is presented to the learner. For multiple-choice questions, it may be enough to simply recognize the best answer from the set of possible choices, which means they may promote more passive processing. In contrast, true—false questions may require a more thorough review of each individual statement, which means they could prompt more constructive processing.

In one study exploring differences in assessment using these two formats on exams in an authentic course context, Couch et al. (2018) varied whether sets of the same four test statements were presented as either a single multiple choice question (containing four response options) or four true—false items. In the multiple-choice format, students were asked which one of the set of four sentences was true (or false), while in the individual format, they made true—false responses for each item separately. Importantly, the true—false items were derived directly from the answer options used in the multiple-choice questions, allowing for a direct comparison across formats. In both question formats, one statement was true and the other three were false. The only difference was that in the true—false condition, students had to make an explicit judgment about every statement, whereas in the multiple-choice version, they only needed to select the correct one. Across samples that were matched in course performance, they found students provided correct answers to the multiple-choice version 67% of the time, compared with only 36% for the same four items presented in true—false format. They concluded that multiple-choice questions may allow students to select an answer without fully processing the meaning or accuracy of the other alternatives, and that multiple-choice tests may overestimate true understanding because students may be able to use partial understanding and passive recognition processes to identify the correct response. When considered from the perspective of practice testing, these results suggest that multiple-choice questions may not always encourage the deeper engagement needed to promote long-term comprehension during practice testing.

On the other hand, there are also reasons why a multiple-choice format could promote more constructive processing than true—false formats. As previously mentioned, comprehension is measured using inference questions. Inference questions require students to go beyond the explicitly stated information in a text and draw conclusions, make connections, or interpret meaning based on context, prior knowledge, and logical reasoning. As such, these questions assess a deeper level of understanding than simple factual recall because they require the reader to infer meaning that is not directly stated. When presented in a multiple-choice format, a benefit is that students know that among the answer choices they are given there is one statement that is true, whereas for all others there is something in there that makes them incorrect. This structure can serve as scaffolding, helping students engage in active reasoning even if their knowledge of the topic is still limited. Work on improving students’ memory for facts has demonstrated that when multiple-choice questions are constructed with competitive alternatives among the answer options, students showed improved recall not only for the directly tested materials but also related concepts tied to competitive elements from the answer alternatives (Alamri & Higham, 2022; Little & Bjork, 2015; Little et al., 2012). Further, under conditions that encourage students to compare and contrast answer choices or examples, this has been found to benefit learning across different contexts, including both mathematics learning and problem-solving as well as text comprehension (Rittle-Johnson & Star, 2007; Schwartz & Bransford, 1998; Williams et al., 2009).

For true—false questions, the truth of each statement must be evaluated independently of the others. Some of each statement can be true with a small, sometimes trivial portion of the statement being false. For learners with limited knowledge who have just started learning about a new topic, it may be extra difficult to identify a trivial detail that is wrong. If the learner feels unsure how to respond to a given statement without being provided any additional information to inform their reasoning, it may be difficult to make a decision and gain any advantage from the practice test. If true—false questions are perceived as too difficult, they might also decrease engagement with the materials and in turn their benefit for learning, while multiple-choice questions may feel easier because the learner is being told which are the possible response options that they must consider. While it is possible to construct true–false questions in ways similar to multiple-choice questions by embedding competitive alternatives within the question stem (Brabec et al., 2021), this approach is probably more of an exception than the norm and may have limited utility depending on the types of comparisons to be encouraged. Incorporating competitive alternatives can be challenging, especially when aiming to promote higher-order reasoning, rather than simple factual contrast. These reasons suggest that greater benefits may be seen from multiple-choice practice tests.

At the same time, one could argue that independence of individual statements is a benefit of the true—false format in that students are prompted to “start over” the evaluative process for each statement as they work through the test. It has been shown there is a tendency for students to mark statements as true more often than false. This “acquiescence bias” has been explained in terms of processing demands where initial acceptance of a statement is automatic while the rejection of a statement as false requires additional cognitive effort (Gilbert et al., 1993). From a constructive processing perspective, if true—false tests prompt the learner to engage in this additional cognitive effort, and to carefully consider each statement in depth, this may prompt the type of reasoning and constructive processing that has been demonstrated to improve comprehension, which suggests greater benefits from practice tests using the true—false format.

Ultimately, these competing considerations underscore that both formats, multiple-choice and true—false, have features that could either help or hinder deeper processing and learning. Any predictions are therefore not based on the assumption that one format is inherently superior to the other, but rather on a broader theoretical argument about how different formats may influence the likelihood of superficial versus constructive engagement during practice testing. Specifically, the central hypothesis is that differences in comprehension outcomes will be driven by the extent to which each test format encourages or discourages superficial responding.

## Current studies

While work on improving comprehension has primarily focused on open-ended generative activities, the primary goal of the present work was to test the question of whether closed-ended practice tests can support comprehension outcomes and whether differences might exist between presenting the practice test in a multiple-choice or true—false format. This question was explored across four experiments. Experiments 1 and 2 compared the two practice test formats to rereading. Experiment 1 tested for differences in comprehension outcomes using a “how-and-why” essay question in Experiment 1, while Experiment 2 used a short-answer (SA) test. After finding advantages for closed-ended practice tests over restudy in the first two experiments, the latter two experiments further explored differences between the two formats. Experiment 3 manipulated feedback on practice tests, while Experiment 4 manipulated text availability during the practice tests.

## Experiment 1

In Experiment 1 the main goal was to test whether completing a practice test using multiple-choice or true—false inference questions would improve comprehension over simply rereading the text for a second time. This study used closed-ended test items in the practice test, but an open-ended how-and-why essay question as the final test. Additionally, it was also of interest whether differences might be seen across the two practice test formats.

### Method

#### Participants

Participants were 116 undergraduates (69% women, mean age = 18.2 years) at a large urban university who received course credit as part of an introductory psychology subject pool. Students identified as 26% Asian, 11% Black/African American, 29% Hispanic/Latinx, and 28% White/Caucasian. This demographic data was collected as part of mass testing at the beginning of the semester. Across all studies, all participants were randomly assigned to condition.

Hinze et al. (2013) reported a medium effect size (*d* = 0.59) for the benefits seen from a constructive processing activity (writing explanations) over rereading. Based on this estimate, an a priori power analysis for a one-way analysis of variance (ANOVA) with three groups (*f* = 0.30) recommended a minimum sample size of 111 to achieve 80% power.

#### Design

The design of the study was between subjects with three conditions: true—false practice test questions, multiple-choice practice test questions, and a reread (no practice test) control condition.

#### Materials

##### Text

The text was from Griffin et al. (2008, 2019) and described the formation and eruption of volcanoes. The text was 1,000 words in length, had a Flesch–Kincaid grade level of 10.6, and a reading ease score of 45.3, placing it in the range of “difficult.” It was written at a level appropriate for college-level readers, and represented a text that students might read as part of a first year college course in geoscience.

##### Practice tests 

The practice tests were adapted from the five multiple-choice inference questions used for this text in Griffin et al. (2019). Because the focus of this work is on comprehension and not just memory for text, only inference questions were used as stimuli (Wiley et al., 2005). Inference questions test connections between ideas and ideas that go beyond that which is explicitly stated in the text. As such, they require access to one’s mental model for the text rather than just verbatim memory. To keep the content of the test questions across conditions as similar as possible, true—false inference questions (20 total for each text) were constructed to map onto the four response options for each of the five multiple-choice questions. The wording of some statements was modified so that the number of true versus false correct was balanced (10 each) in the true—false format. An example of a multiple-choice question and the matching true—false alternatives are presented in Table 1.

**Table 1:** Practice test example question

Multiple-choice	True—false
What causes violent eruptions?	
a) Fluid magmas that are low in silica	Fluid magmas that are low in silica do not erupt violently. (T)
b) **Magmas that come from melted continental plates**	Magmas that come from melted continental plates lead to violent eruptions. (T)
c) Magmas that are high in basalt	Magmas high in basalt cause violent eruptions. (F)
d) Magmas that come from melted oceanic plates	Volcanoes filled with magmas that come from melted oceanic plates erupt violently. (F)

#### Procedure

The experiment was fully computerized and administered online in two half-hour sessions through Qualtrics. Instructions and procedure were based primarily on Experiment 3 by Hinze et al. (2013). For Part 1, all participants were told they would read a short text about volcanic eruptions and that they would be tested on their understanding after 48 h. All participants were also told they would perform a short activity to help them prepare for the final test and that for many of these activities they could not revisit the original text later. After reading, participants engaged in their assigned practice test or rereading. Participants received the link to access Part 2 of the study 48 h following completion for Part 1. In Part 2, comprehension of the text was assessed with a final test consisting of a “how-and-why” essay question. The exact instructions were as follows:

You will now be tested on your understanding of the text you read during Part 1 of the study. Based on the information that you read in the text about volcanoes, explain why and how volcanic eruptions occur. Please write at least five full sentences.

All activities were self-paced. No feedback was provided for performance on the practice test. The text was not available either during the practice tests or the final test. The final essay task was the same across all conditions. Participants’ ACT scores and self-reported prior topic knowledge were collected at the end of the experimental session. Prior topic knowledge was rated on a 0–10 scale, where 0 = *none* and 10 = *a lot*.

#### Scoring

Responses on the essay task were scored on a scale of 0–5 using rubrics based on prior work on these texts (Sanchez & Wiley, 2006; Wiley et al., 2009). To create the rubric, the explanatory text was analyzed for its underling causal model. Each response was scored for the presence of five correct causal concepts. To provide evidence for the reliability of the hand-coded quality scores, open-ended responses were also coded in a second way using latent semantic analysis (LSA; Landauer, et al., 1998) where the semantic overlap between a constructed model response and each participant response was computed. To do this, participant responses were corrected for misspellings, abbreviations, and to expand contractions. For each participant response, the analysis resulted in a coefficient for the degree of semantic overlap with the model response, with numbers closer to 1 representing a greater degree of semantic overlap. Semantic overlap was computed using a one-to-many, document-to-document analysis using the general reading up to first year college LSA space with maximal factors included. The correlation between the hand-coded quality scores and the LSA scores was positive and significant, *r*(114) = 0.44, *p* < 0.001. The hand-coded quality scores were used for analyses.

### Results

A check for random assignment was completed prior to analyses. No significant differences were found in self-reported prior knowledge, *F*(2, 113) = 1.33, *MSE* = 3.58, *p* = 0.267; or ACT scores, *F*(2, 107) = 0.807, *MSE* = 19.02, *p* = 0.449 across conditions. Summary descriptives are provided in Table 2. No significant difference was seen in time spent on the practice activity across conditions overall, *F*(2, 113) = 2.59, *MSE* = 112,482, *p* = 0.080, or between the two testing conditions, *t*(69) = 0.58, *p* = 0.561.

**Table 2:** Descriptive summary across conditions in Experiment 1

	Multiple-choice		True—false		Reread	
	*M*	*SD*	*M*	*SD*	*M*	*SD*
Prior knowledge	3.09	1.88	2.83	1.42	3.51	2.20
ACT	24.8	4.34	23.7	3.89	24.9	4.79
Time spent on practice activity	218	218	246	186	376	474

*Note. *Prior knowledge was reported on a 0–10 scale, where 0 = none and 10 = a lot. The ACT is a standardized assessment for college preparedness. Time spent on practice activities is reported in seconds

#### Practice test performance

Average proportion correct on the practice test was significantly above chance for both multiple choice, *M* = 0.42 (*SD* = 0.23), *t*(34) = 4.42, *p* < 0.001, and true—false formats, *M* = 0.61 (*SD* = 0.13), *t*(35) = 5.25, *p* < 0.001.

#### Comprehension outcomes

**Fig. 1:**
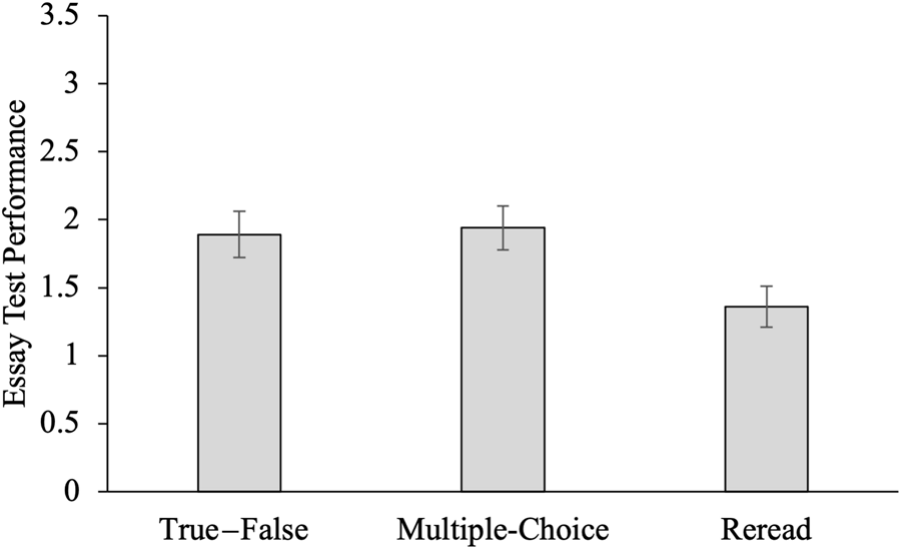
Mean essay test score across practice test and reread conditions in Experiment 1. Error bars represent ± 1 *SEM*

The comprehension outcomes for each condition (mean performance on the final essay task) are shown in Fig. 1.

To test whether differences existed between the three conditions in comprehension outcomes, performance on the essay task was evaluated in a one-way ANOVA. The effect of condition was significant, *F*(2, 113) = 4.31, *MSE* = 1.01, *p* = 0.016, η_p_^2^ = 0.071. Planned comparisons using Tukey corrections revealed that taking a practice test in both multiple-choice, *t*(113) = 2.38, *p* = 0.050, *d* = 0.53, and true—false, *t*(113) = 2.60, *p* = 0.028, *d* = 0.59, formats resulted in better comprehension outcomes than did rereading the text. Differences between the two practice test formats were not significant, *t*(113) = 0.23, *p* = 0.972, *d* = 0.05.

### Discussion

Results from Experiment 1 show a significant testing effect for both the multiple-choice and true—false formats of the practice test. While it was encouraging to see evidence that practice testing with inference questions may indeed improve comprehension outcomes beyond simply rereading a text, average final test performance was low even in the higher performing testing conditions. For the final essay task, it should be noted that a “how-and-why” open-ended prompt can be challenging in that it provides minimal direction for what and how much information is necessary for a “good”’ or complete response. Providing additional scaffolding to clarify what the question is asking students to explain could help reduce this variability and ensure more consistent interpretation of the task which should help increase the sensitivity of the measure. Thus, in Experiment 2 the final essay task was changed to a short-answer (SA) format. The SA test was constructed to consist of five questions, one each for the five causal concepts that made up the coding rubric for the how-and-why essay task in Experiment 1.

## Experiment 2

Experiment 2 was designed to conceptually replicate Experiment 1, comparing multiple-choice and true—false practice test conditions to a rereading control. Materials for this study were the same as those in Experiment 1, with the exception that the final learning assessment was changed from a single open-ended how-and-why essay prompt to an SA format. This SA test was constructed to consist of five questions, one each for the five causal concepts that made up the coding rubric for the how-and-why essay test that was previously used. Testing students’ comprehension using more specific questions was meant to increase the sensitivity of the measure by prompting more complete responses touching on the different causal elements. It was expected that students in both multiple-choice and true—false practice testing conditions would perform better on the final SA assessment than those assigned to the rereading condition. Moreover, with greater sensitivity from the SA assessment, it was also expected that differences between the two testing formats could emerge where either true—false or multiple-choice formats might provide added benefits relative to the other.

### Method

#### Participants

Participants were 114 undergraduates (64% women, mean age = 19.4 years) from the same population as Experiment 1. The target sample size was set to be the same as in Experiment 1. The sample’s self-reported racial profile was 34% Asian, 4% Black/African American, 36% Hispanic, and 26% White/Caucasian. 

#### Design

The design of the study was between subjects with three conditions: true—false practice test questions, multiple-choice practice test questions, and a reread (no practice test) control condition.

#### Materials

Materials including the text and practice tests were the same as in Experiment 1. The final comprehension assessment was changed from an essay to an SA format. The SA test consisted of five questions, one each for the five causal concepts that made up the coding rubric for the how-and-why essay test that was previously used in Experiment 1 (e.g., “How and why does magma composition matter for volcanic eruptions?”). Responses on the SA assessment were scored as 0 (*incorrect*), 0.5 (*partially correct but incomplete*), or 1 (*correct*).

#### Procedure

The procedure was the same as in Experiment 1.

### Results

Prior to analyses a check for random assignment was completed. As shown in Table 3, differences across conditions did not reach significance for either self-reported prior knowledge, *F*(2, 111) = 1.54, *MSE* = 2.80, *p* = 0.218; or ACT scores, *F*(2, 93) = 1.37, *MSE* = 19.00, *p* = 0.259. No differences were seen in the amount of time that participants spent on the practice activity in the study across conditions, *F*(2, 111) = 0.67, *MSE* = 300,114, *p* = 0.514.

**Table 3:** Descriptive summary across conditions in Experiment 2

	Multiple-choice		True–false		Reread	
	*M*	*SD*	*M*	*SD*	*M*	*SD*
Prior knowledge	3.47	1.89	2.92	1.59	2.84	1.53
ACT	24.9	4.08	23.6	4.80	23.0	4.08
Time spent on practice activity	220	297	301	232	368	864

*Note. *Prior knowledge was reported on a 0–10 scale, where 0 = none and 10 = a lot. The ACT is a standardized assessment for college preparedness. Time spent on practice activities is reported in seconds

#### Practice test performance

Average proportion correct on the practice test was similar to Experiment 1 and significantly above chance for both multiple choice, *M* = 0.45 (*SD* = 0.25), *t*(35) = 4.89, *p* < 0.001, and true—false formats, *M* = 0.63 (*SD* = 0.14), *t*(39) = 6.09, *p* < 0.001.

#### Comprehension outcomes

**Fig. 2:**
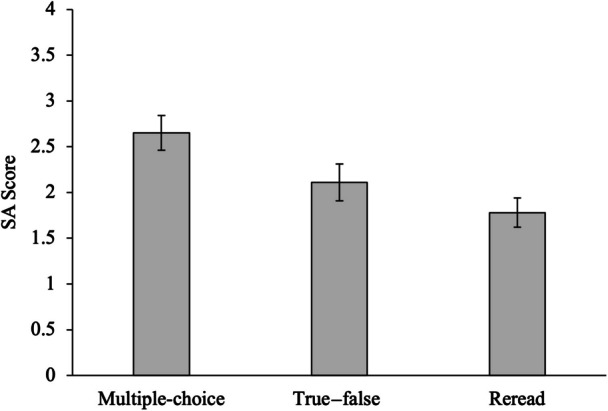
Short-answer test performance across practice test and reread conditions in Experiment 2. Error bars represent ± 1 *SEM*

The comprehension outcomes for each condition (mean performance on the final SA assessment) are shown in Fig. 2. To test whether differences existed between the three conditions in supporting comprehension, performance on the final SA assessment was evaluated in a one-way ANOVA. The effect of condition was significant, *F*(2, 111) = 5.57, *MSE* = 1.29, *p* = 0.005, η_p_^2^ = 0.091.

Planned comparisons using Tukey corrections indicated that multiple-choice practice testing resulted in significantly better performance on the SA test than rereading the text, *t*(111) = 3.31, *p* = 0.004, *d* = 0.77. The difference between rereading and taking a true—false practice test was not found to be significant, *t*(111) = 1.31, *p* = 0.395, *d* = 0.30, nor was the direct comparison between multiple-choice and true—false formats, *t*(111) = 2.07, *p* = 0.101, *d* = 0.48. Thus, results show a significant testing effect only for the multiple-choice practice test. In contrast, taking the true—false practice test did not result in significantly better comprehension outcomes than rereading the text.

Additionally, the data were examined by estimating a Bayes factor using Jamovi’s built-in Bayesian model comparison procedures, which integrate over default Cauchy prior distributions. The one-way ANOVA examining the effect of condition on SA test performance yielded a Bayes factor of 7.39, indicating that the data are 7.39 times more likely under the alternative hypothesis (i.e., that condition has an effect) than under the null hypothesis. This represents positive or substantial evidence. The comparison between the multiple-choice practice test format and rereading yielded a Bayes factor of 38.33, providing strong evidence in favor of the multiple-choice format over rereading. In contrast, the comparison between the true—false format and rereading resulted in a Bayes factor of 0.49, suggesting that the data are 2.04 times more likely under the null hypothesis (i.e., favoring no difference between true—false practice and rereading) for this comparison. Finally, comparing the true–false format directly with the multiple-choice practice format produced a Bayes factor of 1.24, indicating only weak or anecdotal evidence for a difference in comprehension outcomes between the two test formats in this study (Kass & Raftery, 1995).

### Discussion

Results from Experiment 2 show a significant testing effect only for the multiple-choice practice test. In contrast to multiple-choice, the true—false version of the practice test did not result in significantly better comprehension outcomes than rereading the text. To explain these findings in favor of multiple-choice practice tests, it is important to consider how this question format may engage learners differently than a true—false practice test. In multiple-choice questions learners are given a set of possible answer choices side by side. Even when they may not recognize the correct answer right away, they do know that amongst the answer choices they are given only one can be correct whereas all others must be false. Thus, to select what they think is the best answer learners may engage in some form of reasoning. Or they may engage in comparing and contrasting between the choices which has been shown to benefit learning in other contexts (Rittle-Johnson & Star, 2007; Schwartz & Bransford, 1998; Williams et al., 2009). 

In contrast, for true—false questions no additional information is provided beyond the very statement a learner is being asked to evaluate. Especially during early stages of learning, this can make it difficult for learners to draw upon relevant details or examples to arrive at a definitive conclusion regarding a statement’s validity. While there remains a 50% chance of guessing the answer correctly, random guessing is unlikely to contribute to persistent learning gains as it does not involve the cognitive effort or deliberate reasoning that may be necessary to update, elaborate, or improve one’s mental model (Hinze et al., 2013). To benefit from true—false testing, readers may require additional support—such as feedback on the practice test to help them learn from it, or access to the text during the test to support the reasoning needed to verify or reject individual statements. Experiment 3 was designed to examine the effects of feedback, while Experiment 4 focused on the role of text availability during practice.

## Experiment 3

While the previous experiments show that closed-ended practice testing can improve comprehension outcomes, the magnitude of these effects was modest. Further, when the final test was changed from essays to more direct short answer questions, findings suggested that only multiple-choice practice tests improved comprehension compared with rereading while a matched true—false practice test did not. Adding feedback may be one way to enhance the effectiveness of closed-ended practice tests. As past research has shown, feedback can help students to correct errors and maintain correct responses. However, the effectiveness of feedback can vary, with some studies showing no benefits at all (see Bangert-Drowns et al., 1991, for a review). Bangert-Drowns et al. (1991) point out two factors that seem to account for almost all of the variation observed in their meta-analysis. First, it is only when students have an opportunity to self-test prior to receiving feedback on what should be the correct answer that feedback actually benefits their learning. In other words, feedback might only be processed meaningfully when students have attempted answering the question on their own first. Second, the effects of feedback on learning may differ across different types of feedback:

- Knowledge of results feedback (KOR): signals whether a student’s response was correct or incorrect.
- Knowledge of correct response feedback (KCR): presents the correct answer to the student.
- Elaborative feedback (EF): presents the correct answer to a student along with an explanation for why it is correct.

Past research has shown that feedback is more effective when it includes the correct answer, presumably because feedback is needed to correct errors and reinforce correct answers, especially when there is some level of uncertainty over what is correct. However, simply indicating whether a student’s response is correct or incorrect (KOR feedback) has been demonstrated to be much less effective than KCR or EF feedback. Under conditions when the goal is not just memory for the correct answer, but deeper understanding of the concepts tested during practice, EF has been found to be most effective (Butler et al., 2013). In Experiment 3, it was of interest to test whether adding feedback (KCR or EF) would improve understanding from closed-ended practice tests as compared with a no-feedback context for a multiple-choice and true—false practice test.

### Method

#### Participants

 Participants were 297 undergraduates (69% women, mean age = 19.1 years) from the same population as prior studies . The self-reported racial profile of the sample was 17% Asian, 7% Black/African American, 43% Hispanic, 22% White/Caucasian, and 12% Other. 

To estimate the required sample size for this experiment, it was necessary to consider effects for both feedback and practice test format. In terms of feedback, effect sizes reported for feedback interventions in the literature can be relatively large (Hedges’s *g* = 0.61–0.92; Bangert-Drowns et al., 1991; Swart et al., 2019). The closest study to the present one is Butler et al. (2013) who found an effect size of *f* = 0.30 from feedback on practice tests. In terms of practice test format, in contrast to using an effect size based in comparisons between practice testing and reread control conditions that were used in Experiments 1 and 2, for Experiment 3 the primary focus of comparison was between the two practice test formats: multiple-choice and true—false. The closest study to the present one is Hinze et al. (2013, Experiment 3), which compared differences in comprehension outcomes for two different practice test formats (free recall vs. explanation). The effect size for this specific comparison was *f* = 0.23. This effect size is similar to the difference between MC and TF in Experiment 2 of *f* = 0.24. Using a conservative estimate of the smaller expected effect, an a priori power analysis using *f* = 0.20 recommended a minimum sample size of 244 to achieve 80% power in a 2 × 3 design (main effects and interaction). This suggests a sample of 297 was adequately powered to detect feedback and format effects.

#### Design

The design of the study was a 2 (practice test format: multiple-choice, true—false) × 3 (feedback type: knowledge of correct response feedback [KCR], elaborative feedback [EF], no feedback [NF]) between-subjects design with six conditions.

#### Materials

The text, practice test, and final SA comprehension assessment were the same as in Experiment 2. For each of the two practice test formats three versions were created, one with knowledge of correct response (KCR) feedback that indicated whether a student’s answer was correct or not along with the correct answer, and another with elaborative feedback (EF), which was identical to KCR but in addition to stating the correct answer also provided an explanation why that answer was correct using sentences copied from the text (see Tables 4 and 5).

**Table 4:** Examples of KCR for the two practice testing formats in Experiment 3

	Multiple-choice	True—false
Knowledge of response feedback (KCR)	You selected: *A* The correct answer is: *B* What causes violent eruptions? A. Fluid magmas that are low in silica B. Magmas that come from melted continental plates C. Magmas that are high in basalt D. Magmas that come from melted oceanic plates	You selected: *F* The correct answer is: *T* Magmas that come from melted continental plates lead to violent eruptions ⚬ T ⚬ F You selected: *T* The correct answer is: *T* Fluid magmas that are low in silica do not erupt violently ⚬ T ⚬ F You selected: *F* The correct answer is: *F* agmas high in basalt cause violent eruptions ⚬ T ⚬ F You selected: *T* The correct answer is: *F* Volcanoes filled with magmas that come from melted oceanic plates erupt violently ⚬ T ⚬ F

**Table 5:** Examples of EF for the two practice testing formats in Experiment 3

	Multiple-choice	True—false
Elaborative feedback (EF)	You selected: *A* The correct answer is: *B* What causes violent eruptions? A. Fluid magmas that are low in silica B. Magmas that come from melted continental plates C. Magmas that are high in basalt D. Magmas that come from melted oceanic plates **Explanation:** *Magmas that come from continental plates are high in silica. When there is more silica in the magma eruptions tend to be more explosive*	You selected: *F* The correct answer is: *T* Magmas that come from melted continental plates lead to violent eruptions ⚬ T ⚬ F You selected: *T* The correct answer is: *T* Fluid magmas that are low in silica do not erupt violently ⚬ T ⚬ F You selected: *F* The correct answer is: *F* Magmas high in basalt cause violent eruptions ⚬ T ⚬ F You selected: *T* The correct answer is: *F* Volcanoes filled with magmas that come from melted oceanic plates erupt violently ⚬ T ⚬ F **Explanation:** *Magmas that come from continental plates are high in silica. When there is more silica in the magma eruptions tend to be more explosive*

For each of the five multiple-choice questions, feedback was displayed on a separate page. Because true—false items were created to match the four multiple-choice response options, for the purposes of feedback equivalency across conditions, the true—false items were clustered in these groups of four. The explanations included as part of EF were identical across multiple-choice and true—false conditions. To ensure that participants would have to engage with the feedback at least on some level, they were required to click on the correct response for each question before being able to advance to the next page. For both practice testing conditions there also was a no-feedback control condition (NF).

#### Procedure

The procedure was similar to Experiments 1 and 2. For those in the feedback conditions, the feedback was provided following the practice test. To ensure that participants attended to the feedback they were instructed to click the correct answer before being allowed to continue.

### Results

A check for random assignment to condition was completed for ACT scores, and prior knowledge ratings. For ACT scores a 2 (practice test format: multiple-choice vs. true—false) × 3 (feedback type: EF vs. KCR vs. NF) ANOVA showed no main effects for either test, *F*(1, 257) = 0.18, *MSE* = 21.64, *p* = 0.668, or feedback conditions, *F*(2, 257) = 0.13, *MSE* = 21.64, *p* = 0.883 and no interaction, *F*(2, 257) = 0.28, *MSE* = 21.64, *p* = 0.753. Likewise, for prior knowledge ratings there also was no main effect for either feedback conditions, *F*(2, 291) = 0.21, *MSE* = 3.61, *p* = 0.809, or test format, *F*(1, 291) = 3.12, *MSE* = 3.61, *p* = 0.079, and no interaction, *F*(2, 291) = 0.22, *MSE* = 3.61, *p* = 0.802. Summary statistics are presented in Table 6. No differences were seen in the amount of time that participants spent on the practice activity across conditions, *F*(1, 291) = 0.20, *MSE* = 107,227, *p* = 0.653. In the feedback conditions, the average time spent on feedback was generally less than 20 s per feedback screen for both KCR (*M* = 11 s, *SD* = 11) and EF (*M* = 17 s, *SD* = 18) conditions.

**Table 6:** Descriptive summary and practice test performance across conditions in Experiment 3

	True—false			Multiple-choice		
	NF *M*(*SD*)	KCR *M*(*SD*)	EF *M*(*SD*)	NF *M*(*SD*)	KCR *M*(*SD*)	EF *M*(*SD*)
ACT	23.6 (5.13)	23.4 (4.95)	23.7 (4.11)	24.2 (4.58)	23.9 (4.63)	23.4 (4.59)
Prior knowledge	3.20 (2.00)	3.27 (1.78)	3.16 (1.63)	3.80 (2.03)	3.53 (1.92)	3.48 (2.05)
Time spent on practice activity	263 (290)	223 (180)	255 (216)	247 (359)	304 (512)	241 (310)

*Note. *Chance performance on the practice test is 0.50 for the true—false format and 0.25 for the multiple-choice format. Time spent on the practice activity is reported in seconds

#### Practice test performance

Mean performance on the practice test across conditions is shown in Table 7. The average proportion correct on the practice test was significantly above chance across all conditions.

**Table 7:** One-sample *t* tests comparing practice test performance in Experiment 3 to chance levels

	*M*	*SD*	*df*	*t*	*p*
Multiple-choice, no feedback	0.55	0.18	39	10.32	< 0.001
Multiple-choice, KCR	0.48	0.15	50	11.33	< 0.001
Multiple-choice, EF	0.49	0.18	49	9.92	< 0.001
True—false, no feedback	0.69	0.09	50	15.10	< 0.001
True—false, KRC	0.70	0.11	43	11.77	< 0.001
True—false, EF	0.71	0.08	60	19.84	< 0.001

*Note. *Chance-level performance for the multiple-choice test is set at 0.25 and at 0.5 for true—false

#### Comprehension outcomes

**Fig. 3:**
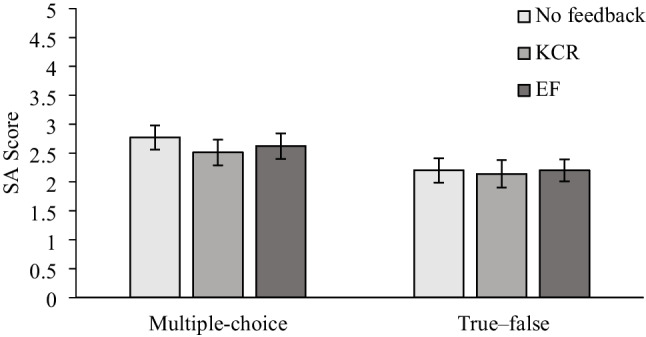
SA test performance across practice test feedback conditions in Experiment 3. Error bars represent ± 1 *SEM*

Mean performance on the SA comprehension test is shown in Fig. 3. A 2 (practice test format: multiple-choice vs. true—false) × 3 (feedback type: EF vs. KCR vs. NF) ANOVA was conducted to compare final test performance across conditions. There was a main effect for practice test format, *F*(1, 291) = 6.78, *MSE* = 2.26, *p* = 0.010, η_p_^2^ = 0.02. Planned comparisons using Tukey corrections indicated that participants who had completed a multiple-choice practice test performed significantly better (*M* = 2.62, *SD* = 1.49) than did those who took a true—false practice test (*M* = 2.18, *SD* = 1.50), *t*(291) = 2.60, *p* = 0.010, *d* = 0.31. There was no main effect for feedback, *F*(2, 291) = 0.61, *MSE* = 2.26, *p* = 0.764, η_p_^2^ < 0.01. There also was no interaction, *F*(2, 291) = 0.27, *MSE* = 2.26, *p* = 0.890, η_p_^2^ < 0.01. As shown in Fig. 3, taking a multiple-choice practice test consistently produced better comprehension outcomes over taking a true—false practice test. Regardless of practice test format or feedback type, adding feedback did not improve learning from the practice tests.

### Discussion

In the two previous experiments, it was shown that practice testing with closed-ended inference questions can improve comprehension over rereading. In Experiment 1, both closed-ended practice formats improved comprehension on final essay tests, while in Experiment 2 only for the multiple-choice and not true—false format of the practice test improved performance on the final short answer tests. The goal of the current study was to test whether adding feedback to closed-ended practice tests would support better comprehension outcomes. Different types of feedback were compared with a no-feedback control condition for both multiple-choice and true—false formats of the practice test.

Contrary to expectations, adding feedback did not improve learning from either of the practice testing formats as compared with a no-feedback version. The type of feedback (KCR vs. EF) also did not matter. Compared with the true—false version of the practice test, the multiple-choice version resulted in better comprehension outcomes. This gap between the two test formats persisted even with feedback, where across conditions the multiple-choice practice test consistently led to better final SA test performance than the true—false practice test.

While the effectiveness of feedback can vary, considering the large number of studies that have shown evidence in support of feedback interventions (Bangert-Drowns et al., 1991; Swart et al., 2019), it was certainly surprising to not see these benefits replicating here. To ensure that participants would have to engage with the feedback at least on some level, they were required to click on the correct response for each question before being able to advance to the next screen. However, given that the average time spent on feedback was generally less than 20 s per feedback screen, participants may not have engaged with the feedback at a level necessary for helping them improve or update their existing mental model (Hinze et al., 2013).

Instead of providing explicit corrections, making the text available during practice testing might be another alternative to support comprehension. By interacting with the text while answering, learners receive implicit feedback, as they can compare their reasoning with the information that is provided in the text. Rather than passively receiving corrections, learners are encouraged to construct meaning through direct engagement with the text. This approach is reminiscent of work on adjunct questions, which suggests that answering questions embedded during reading can help readers to organize their mental model for the ideas presented in a text, to direct their focus on that information which is most relevant to understanding, and to improve their comprehension (Callender & McDaniel, 2007; Hamaker, 1986; Rubio et al., 2022).

## Experiment 4

Experiment 4 was designed to investigate the effects of text availability during practice testing on comprehension outcomes across the two practice test formats. In previous experiments, the multiple-choice version of the practice test led to better comprehension outcomes than a true—false version of the same test. One possible reason for why the multiple-choice format was found to be more effective is that in the multiple-choice format “more” contextual information is provided at the time of test. Among the list of possible answer choices learners know that only one of these options is correct. In contrast, in a true—false format learners have to decide on the validity of each individual statement they are given where there is less scaffolding provided on which information they might consider in determining their answers. Making the text available during practice testing might increase learner engagement and support reasoning, particularly when answering true—false questions, because learners can search the text for information that would support or falsify the statements they are given. Thus, Experiment 4 aimed to determine whether the advantages of multiple-choice testing over true—false testing might be reduced or reversed under conditions when the text is accessible during practice. It was hypothesized that when the text is available this might increase students constructive processing and reasoning with the materials and thus increase comprehension outcomes.

### Method

#### Participants

A sample of 288 undergraduates (72% women, mean age = 19 years) from from the same population as prior studies. The self-reported racial profile of the sample was 21% Asian, 7% Black/African American, 45% Hispanic, and 21% White/Caucasian. 

To estimate the required sample size for this experiment, it was again necessary to consider effects for both text availability and practice test format. In terms of text availability, the closest study to the present one is Rubio et al. (2022) who found an effect size of *f* = 0.30 comparing adjunct questions answered during reading to a practice test administered postreading. In terms of practice test format, Experiment 3 suggested an effect size of *f* = 0.20 for differences between multiple-choice and true—false practice test formats. Using the estimate of the smaller expected effect, an a priori power analysis using *f* = 0.20 recommended a minimum sample size of 199 to achieve 80% power in a 2 × 2 design (main effects and interaction). This suggests a sample of 288 was adequately powered to detect text availability and practice format effects.

#### Design

The design of the study was a 2 (practice test format: multiple-choice, true—false) × 2 (text availability: text available, no text available during practice test) between-subjects design with four conditions.

#### Materials

Materials were the same as those used in previous experiments.

#### Procedure

The procedure for the two no-text-available practice test conditions matched that of Experiment 2 and the no-feedback control condition in Experiment 3. All participants read the text and following reading engaged in their assigned practice activity that consisted of either a multiple-choice or a true—false practice test. In two other practice test conditions, the text was made available during the practice activity, resulting in a 2 × 2 design. As in previous experiments, the final SA comprehension assessment was completed in a separate session after a 48 h delay. The text was not available to any of the participants during the final SA test.

### Results

Prior to any analyses, a check for random assignment to condition was completed for ACT scores and self-reported prior knowledge. A summary of these sample descriptives is shown in Table 8. A 2 (practice test format: multiple-choice vs. true—false) × 2 (text available: yes vs. no) ANOVA examining ACT scores across conditions did not show significant main effects for either test format, *F*(1, 259) = 0.34, *MSE* = 21.13, *p* = 0.563, text availability, *F*(1, 259) = 1.04, *MSE* = 21.13, *p* = 0.309, or their interaction, *F*(1, 259) = 0.61, *MSE* = 21.13, *p* = 0.437. Similarly, the main effects and interaction were also not significant for prior knowledge scores (all *F* values < 1).

**Table 8:** Descriptive summary across conditions in Experiment 4

	Multiple-choice				True—false			
	No text		Text available		No text		Text available	
	*M*	*SD*	*M*	*SD*	*M*	*SD*	*M*	*SD*
Prior knowledge	3.35	1.97	3.21	2.02	3.20	1.94	3.23	1.91
ACT	23.5	5.02	22.5	4.88	22.7	4.12	22.6	4.23
Time spent on practice activity	672	554	1531	1077	678	637	1678	972

*Note.* Prior knowledge was reported on a 0–10 scale, where 0 = none and 10 = a lot. The ACT is a standardized assessment for college preparedness. Time spent on the practice activity is reported in seconds

No significant differences were seen in the amount of time that participants spent on the practice activity in the study across testing conditions, *F*(1, 284) = 0.59, *MSE* = 714,046, *p* = 0.441, however participants did spend longer when the text was available, *F*(1, 284) = 87.05, *MSE* = 714,046, *p* < 0.001.

#### Practice test performance

As in previous experiments, performance on the practice tests was significantly above chance for multiple-choice and true—false formats both when the text was available and when it was not (Table 9). The procedural change of presenting the text during the practice tests did improve performance in both multiple-choice, *t*(145) = 2.53, *p* = 0.01, *d* = 0.42, and true—false format conditions, *t*(139) = 4.83, *p* < 0.001, *d* = 0.82.

**Table 9:** One-sample *t* tests comparing practice test performance in Experiment 4 to chance levels

	*M*	*SD*	*df*	*t*	*p*
Multiple-choice, no text available	0.46	0.25	69	8.32	< 0.001
Multiple-choice, text available	0.57	0.28	70	15.10	< 0.001
True—false, no text available	0.62	0.12	70	7.13	< 0.001
True—false, text available	0.72	0.12	75	10.01	< 0.001

*Note.* Chance level performance for the multiple-choice test is set at 0.25 and at 0.5 for true—false

#### Comprehension outcomes

**Fig. 4:**
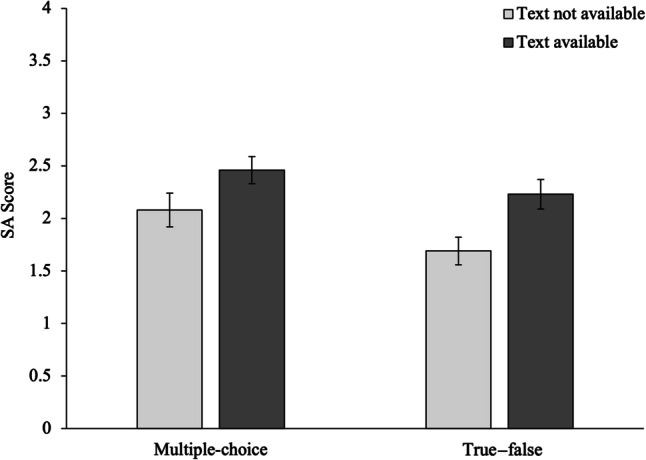
Comprehension test performance across practice test and text availability conditions in Experiment 4. Error bars represent ± 1 *SEM*

The comprehension outcomes for each condition (mean performance on the final SA assessment) are shown in Fig. 4. To examine the effects of practice condition on comprehension, a 2 × 2 ANOVA was conducted, with test format and text availability serving as independent variables. There was a significant main effect for practice test format, *F*(1, 284) = 5.03, *MSE* = 1.40, *p* = 0.026, η_p_^2^ = 0.02. As shown in Fig. 4 multiple-choice practice tests resulted in better performance on the final SA comprehension assessment than the true—false version of the practice test. The main effect for text availability was also significant, *F*(1, 284) = 15.31, *MSE* = 1.40, *p* = 0.001, η_p_^2^ = 0.04. Those who completed the practice test with the text available performed better on the final comprehension test compared with those who did not have access to the text during practice. The interaction between practice test format and text availability was not significant, *F*(1, 284) = 0.37, *MSE* = 1.40, *p* = 0.542, η_p_^2^ = 0.001. The benefits from multiple-choice practice test formats over true—false formats were not eliminated when the text was available during practice testing.

### Discussion

Results from Experiment 4 again show a significant benefit for the multiple-choice over the true—false practice test format in terms of performance on the final SA comprehension assessment. Performance on both versions of the practice test improved when the text was available during practice, which translated to better performance also on the final SA test across both conditions. Still, the overall benefit for the multiple-choice format was sustained even under text-available conditions. Considering that learners were exposed to the same content for the multiple-choice and true—false versions of the practice test, it appears that question presentation in some way impacts learner engagement during practice.

## General discussion

Across four experiments, the primary goal of the present work was to test the question whether closed-ended practice tests can support comprehension outcomes and whether differences might exist between presenting the practice test in a multiple-choice or true—false format. In Experiments 1 and 2, multiple-choice and true–false practice test conditions were compared with a control condition, where instead of answering practice questions, learners reread the texts for a second time. In Experiment 1, both the multiple-choice and true—false version of the test resulted in better comprehension outcomes as measured by their performance on a “how-and-why” essay task. Due to overall low scores in Experiment 1, in Experiment 2 the comprehension measure was changed to an SA test to provide more explicit instructions on how learner’s understanding was being tested. The goal was that these clearer prompts would help students better understand what to include in their responses to perform well. If a more specific prompt could increase participants’ understanding of what to write about in their responses this, in turn, was expected to increase the sensitivity of the comprehension measure. For the SA test, only the multiple-choice practice test resulted in better comprehension outcomes compared with rereading whereas the true—false version of the test did not. This SA test was used for the remainder of the studies. A benefit of multiple-choice practice over true—false practice on comprehension outcomes was consistently observed across Experiments 2, 3, and 4.

In Experiment 3, feedback was added to the practice test. Contrary to expectations, including feedback did not improve comprehension beyond what was observed for the no-feedback control condition. Regardless of the type of feedback that was added, if any, the multiple-choice version of the practice test resulted in better comprehension than the true—false version.

Experiment 4 manipulated text availability during the practice test. While adding explicit feedback and explanations in Experiment 3 did not provide an additive benefit, it was hypothesized that when the text is available this might increase students’ constructive processing and reasoning with the materials. Moreover, when the text is available, this could potentially reduce or even reverse the differences seen between the multiple-choice and true—false formats. Results showed that overall, students’ comprehension was better when the text was available during practice testing than when it was not, for both formats of the practice test. However, those who completed the multiple-choice version of the practice test performed better on the final SA comprehension measure than those who completed the true—false version, both when the text was not available but also when it was.

Taken together, this work extends prior work showing benefits in comprehension from practice tests that require students to construct a response, and replicates earlier findings that practice tests with closed-ended questions can also support comprehension. However, consistent with both Experiments 2 and 3, it was also shown that question format can matter for closed-ended practice tests where the multiple-choice version was more effective than the true—false version of the same test in supporting comprehension. While most work on improving comprehension has focused on open-ended generative activities, it is encouraging that closed-ended practice tests were seen to also benefit comprehension. Unlike open-ended alternatives, closed-ended practice tests offer the advantage of being easily incorporated into various learning environments, requiring less time, and facilitating straightforward grading—all of which are concerns previously expressed by educators regarding the implementation of practice testing in the classroom (Roediger & Karpicke, 2006).

Multiple-choice questions have sometimes been criticized for allowing passive responding because answers are provided and one must be correct (Ben-Simon et al. 1997; McAllister & Guidice, 2012; Paxton, 2000). However, the present work suggests, when used as a tool to support comprehension, and especially during early stages of learning, those same characteristics may provide an advantage. For multiple-choice questions, the answer choices themselves include some hints with regards to what information the student must consider and which all cannot be true at once. Also, in a multiple-choice format, all possible answer choices are presented side by side. By design, this form of question presentation may encourage comparison processes among competing alternatives (Alamri & Higham, 2022; Little & Bjork, 2015; Little et al., 2012) in the learner and in turn, they may engage in more active reasoning processes that helps them organize their mental model for the text. During early stages of learning when students’ mental models are often faulty and/or incomplete, multiple-choice items may help students to access relevant information more so than true—false formats. A limitation of Experiments 3 and 4 is there was no restudy control, so one cannot determine whether comprehension is improved by multiple-choice or harmed by true—false practice. But the same difference was found across multiple studies, and both showed the consistent advantage from the multiple choice format over true—false.

The present work identifies important constraints and subtle nuances in question design that can influence whether benefits for learning may be observed from practice testing. It should be emphasized that in this study learners were exposed to the same content across the two question-type formats. The true—false inference questions (20 total for each text) were specifically constructed to map onto the four response options for each of the five multiple-choice questions. Thus, although more work is needed to test the underlying mechanism by which multiple-choice testing was found to be more effective than using a true—false format, it appears to be differences in presentation rather than content affecting learners’ engagement with these questions which translated into performance differences on the final SA assessment. Another limitation of the current work is that all data were collected online so that no participant observation was possible as they engaged in the reading, practice, and assessment tasks. While some timing data are reported in this manuscript, timing estimates may not be reliable as the source of timing variation is unknown and could result from a multitude of source that can include both on and off-task behaviors that participants may have engaged in while completing the task in a remote setting. In a more controlled environment, it might be easier to isolate how participants engagement with the materials may differ across practice test conditions. Trace methods such as think-aloud protocols or eye tracking might help to identify how the two test formats cause differences in processing during the practice test activities, and could test whether active comparison among responses is related to better learning from the multiple-choice formats.

It is also important to note that the final test questions used in these studies to test for learning and understanding of the text and were not the same questions used in the practice tests. Although many testing effects have been reported using repeated presentation of the same questions, this type of result may only show that very specific information (the exact question and answer) has been learned. In contrast, when new questions are used this helps to ensure that any benefits from practice tests are not simply the result of prior exposure to specific questions and responses, and that the answers that the learner needs to generate about each process described in the text provide a measure of quality of their situation model or mental model constructed from the text, reflecting the learner’s broader understanding. In the studies presented here, a multiple-choice practice test resulted in better comprehension outcomes than a true—false version.

## Data Availability

The data and code for these experiments are available on OSF (https://osf.io/r98zf/). Materials are available upon request from the authors. The experiments were not preregistered.

## Declarations

### Author note

The authors thank M. Anne Britt and Thomas D. Griffin for their valuable feedback and discussion, and research assistants Jen Eng, Gabriella Rodriguez, and Meaghan Schmugge for assistance with scoring. Data from Experiment 1 were previously reported in Hildenbrand and Wiley (2021). Portions of this work were completed as part of the first author’s master’s thesis.

### Open practices statement

The data for this experiment is available on OSF (https://osf.io/r98zf/). Materials are available upon request to the authors. The experiments were not preregistered.
